# Synchrotron microtomography reveals insights into the degradation kinetics of bio-degradable coronary magnesium scaffolds

**DOI:** 10.1016/j.bioactmat.2023.09.008

**Published:** 2023-09-23

**Authors:** Roman Menze, Bernhard Hesse, Maciej Kusmierczuk, Duote Chen, Timm Weitkamp, Stephanie Bettink, Bruno Scheller

**Affiliations:** a MeKo Manufacturing e.K., Im Kirchenfelde 12-14, 31157, Sarstedt, Germany; b Xploraytion GmbH, Bismarckstr. 10-12, 10625, Berlin, Germany; c Synchrotron SOLEIL, L'Orme des Merisiers, Départementale 128, 91190, Saint-Aubin, France; d InnoRa GmbH, Robert-Koch-Platz 4, 10115, Berlin, Germany; e Universität des Saarlandes, Campus Homburg, 66421, Homburg, Germany

**Keywords:** Resoloy, Magnesium scaffold, DCB, Paclitaxel, Degradation, Inflammation, Artificial intelligence

## Abstract

Bioresorbable magnesium scaffolds are a promising future treatment option for coronary artery stenosis, especially for young adults. Due to the degradation of these scaffolds (<1 year), long-term device-related clinical events could be reduced compared to treatments with conventional drug eluting stents. First clinical trials indicate a return of vasomotion after one year, which may be associated with improved long-term clinical outcomes. However, even after decades of development, the degradation process, ideal degradation time and biological response in vivo are still not fully understood.

The present study investigates the in vivo degradation of magnesium scaffolds in the coronary arteries of pigs influenced by different strut thicknesses and the presence of antiproliferative drugs. Due to high 3D image contrast of synchrotron-based micro-CT with phase contrast (SR-μCT), a qualitative and quantitative evaluation of the degradation morphology of magnesium scaffolds was obtained. For the segmentation of the μCT images a convolutional network architecture (U-net) was exploited, demonstrating the huge potential of merging high resolution SR-μCT with deep learning (DL) supported data analysis.

In total, 30 scaffolds, made of the rare earth alloy Resoloy®, with different strut designs were implanted into the coronary arteries of 10 domestic pigs for 28 days using drug-coated or uncoated angioplasty balloons for post-dilatation. The degradation morphology was analyzed using scanning electron microscopy, energy dispersive x-ray spectroscopy and SR-μCT. The data from these methods were then related to data from angiography, optical coherence tomography and histology.

A thinner strut size (95 vs. 130 μm) and the presence of paclitaxel indicated a slower degradation rate at 28 d in vivo, which positively influences the late lumen loss (0.5 and 0.6 mm vs. 1.0 and 1.1 mm) and recoil values (0 and 1.7% vs. 6.1 and 22%).

## 1 Introduction

Magnesium scaffolds appear very attractive for future care of vascular diseases due to their good biocompatibility, superior mechanical strength and faster degradation rates compared to recent polymeric scaffolds. The CE-certified magnesium scaffold Magmaris (Biotronik AG, Bülach, Switzerland) and its predecessors showed not only excellent clinical outcomes in first studies but also some promising features like *anti*-thrombogenicity and *anti*-restenotic abilities [[1], [2], [3], [4], [5]]. First preclinical data of Magmaris's successor, DREAMS-3G, showed even longer scaffolding time, which might be beneficial in terms of recoil of the scaffold and accompanying late lumen loss (LLL) [6].

Resorbable scaffolds could improve the treatment of vascular diseases and congenital heart disease of infants, children, and young adults as they do not hinder natural vessel growth and vasomotion after degradation. Furthermore, this technique offers the possibility of a re-intervention at the same location, whereas a non-resorbable metal stent has to be post-dilated or surgically removed [[7], [8], [9]].

Although resorbable magnesium scaffolds have been investigated for the past two decades, there is still a need for better understanding of their in vitro and in vivo degradation mechanisms. The rate and homogeneity of degradation are important to understand and predict the biological tissue response, the recoil, the un-caging of the vessel and the timeline for complete resorption of the scaffold. In the past, this information was addressed in 2D by planimetric degradation analysis, scanning electron microscopy (SEM), energy dispersive X-ray spectroscopy (EDX) and in 3D mainly through laboratory micro-CT analysis. While analyses based on 2D imaging methods can suffer from the impact of sample preparation or surface artefacts, conventional 3D laboratory μCT suffers from limited material contrast and spatial resolution and performs poorly in visualizing soft tissue together with the scaffold [2,[10], [11], [12]]. Laboratory-based μCT with phase contrast can combine high contrast with high resolution, but is not widely available, and image acquisition is very slow [13].

Synchrotron-based μCT (SR-μCT) with phase contrast is becoming increasingly popular and available to study biomaterials, such as degradable Mg implants or membranes [14,15]. SR-μCT provides high image quality due to the high intensity and the coherence of the X-ray beams produced by synchrotron light sources. The high coherence allows for phase contrast imaging which is about 1–2 orders more sensitive to mass density modulations than conventional absorption contrast μCT imaging [16]. The resulting 3D volumes of one single sample obtained through SR-μCT can easily exceed hundreds of GB. In the case of resorbable magnesium scaffolds a quantitative analysis of the corroded and metallic phases requires the segmentation of the different material phases. However, such segmentation can be challenging and time consuming because of the complexity of the corrosion process resulting in complex corrosion fronts and corrosion phases with material densities close to the initial metallic phase. Recently, convolutional networks were successfully used for 3D image segmentation of various materials, including degradable magnesium bone implants [[17], [18], [19], [20]].

To the best of our knowledge, the present study is the first one that quantitatively shows the degradation of explanted coronary scaffolds by SR-μCT. For the data analysis a convolutional network architecture (U-net) was exploited, demonstrating the huge potential of merging high resolution SR-μCT with deep-learning (DL) techniques for data analysis. In the present study, we aim to evaluate the influence of wall thickness, alloying and post-dilatation with either plain old balloon angioplasty (POBA) or a drug-coated balloon (DCB) on the degradation behavior and biological response of scaffolds made of Resoloy®. Both, thinner struts and the use of anti-proliferative drugs reduce restenosis and re-interventions [6,21,22].

Resoloy is a patented bioresorbable rare-earth-containing magnesium alloy. Due to its high strength and ductility, it is suited for use in vascular scaffolds with smaller wall thickness and higher radial strength than polymeric scaffolds. Furthermore, Resoloy scaffolds do not have some of the constraints of polymeric scaffolds, such as stepped inflation during expansion or a slow degradation time, which might provoke poor interventional results [[23], [24], [25]].

## 2 Materials and methods

### 2.1 Scaffolds and balloon catheters

The scaffolds, consisting of Resoloy as base material (Table 1), were laser cut from an extruded tube (diameter: 1.8, wall thickness: 0.16 mm). Each specimen underwent electropolishing, heat treatment and passivation in hydrofluoric acid. The scaffolds were 15 mm long, with a wall thickness of either 130 or 95 μm and a strut width of either 130 or 110 μm. The design consisted of eight crowns and twelve links.

**Table 1:** Alloy composition and mechanical characteristics of Resoloy.

Alloy composition [w.%]	
Dysprosium	5–10
Neodymium	1
Zinc	1
Zirconium	0.2
Magnesium	Balance
Ultimate tensile strength [MPa]	280 +- 10
Break elongation [%]	32 +- 2

Scaffolds were crimped on semi compliant balloon-catheters (diameter: 2.75 mm, length: 18 mm) with a resulting crossing profile <1.2 mm. Post-dilatation was performed with either a paclitaxel DCB (Acotec Scientific, AcoArt Litos, Beijing, China) or POBA (Terumo, Ryurei (RX), Tokyo, Japan) with a diameter of 3.0 mm and 40 mm length. The characteristics of the four groups tested are listed in Table 2.

**Table 2:** Scaffold groups and sample sizes.

Group	G130_10%	G95_10%_POBA	G95_10%_DCB	G95_5%_DCB
scaffold wall thickness	130 μm	95 μm	95 μm	95 μm
alloy	10Dy1Nd1Zn0·2Zr	10Dy1Nd1Zn0·2Zr	10Dy1Nd1Zn0·2Zr	5Dy1Nd1Zn0·2Zr
post-dilatation	none	POBA	DCB	DCB
total sample size	n = 12	n = 6	n = 6	n = 6
SRμ-CT sample size	n = 3	n = 3	n = 3	n = 3
histology sample size	n = 12	n = 6	n = 6	n = 6
OCT sample size	n = 12	n = 5	n = 5	n = 5
Angio sample size	n = 12	n = 4	n = 5	n = 5

### 2.2 Animal study

In total, 10 domestic pigs underwent scaffold implantation. In each animal 3 scaffolds were implanted in the coronary arteries (LAD, LCX and RCA) with a balloon-to-artery ratio of 1.1.

Quantitative coronary angiography (QCA) and optical coherence tomography (OCT) were performed to measure the corresponding vessel morphology before and after implantation. This was repeated at follow-up (FUP) 28 days later, which was the end-point of the trial. The QAngio® XA System (Medis Medical Imaging, Leiden, the Netherlands) was used for quantitative analysis of angiograms. The OCT images were recorded using the ILUMIEN™OPTIS™ system (Abbott Laboratories, Chicago, USA) to evaluate proper device deployment after scaffold implantation and at 28 days follow-up. Qualitative and quantitative analyses were done on the ILUMIEN™OPTIS™ offline review workstation.

The LLL and recoil (R) were determined by the following calculations:

LLL=minimallumendiameter(implantation)−minimallumendiameter(FUP)

and

$$R=\frac{m\text{ean}\;\text{lumen}\;\text{diameter}\;\left(\text{implantation}\right)-\text{mean}\;\text{lumen}\;\text{diameter}\;\left(FUP\right)}{\text{mean}\;\text{lumen}\;\text{diameter}\;\left(\text{implantation}\right)}\;x\;100\%\text{.}$$

After euthanasia, the target vessel segments were harvested, fixed in formalin, dried and embedded in methyl methacrylate for histology and μCT. The whole block was used for μCT and three sections of each sample were obtained and analyzed for histology. The inflammation scores were graded according to Kornowski et al. [26].

The tests were performed at the Institute of Medical Technology and Research (IMTR GmbH, Rottmersleben, Germany). All animals received standard care outlined in accordance with the EU Commission Directive 86/609/EEC and the German Animal Protection Act based upon the Animal Ethics Committee approvals (Saxony-Anhalt, Germany).

### 2.3 SEM and EDX

After histology processing, three extant blocks of each group were used for SEM and EDX analysis. The blocks were ground with a 4000 grit silicon carbide abrasive paper and water as a lubricant to generate a smooth surface of the respective cross section. After drying in warm air, they were covered with a thin gold layer using a sputter coater (108 auto, Cressington Scientific Instruments, Watford, UK). Images of struts of each scaffold group were taken using the SEM (Vega 3, Tescan, Brno, Czech Republic) in backscattered-electron (BSE) mode with 20 kV acceleration voltage. EDX elemental mapping of single struts was performed to qualitatively determine the element distribution (Thermo Fisher Scientific, Waltham, Massachusetts, USA).

### 2.4 Synchrotron μCT data collection and reconstruction

X-ray phase contrast microtomography was performed at the beamline ANATOMIX of Synchrotron SOLEIL (Saint-Aubin, France) [27,28]. The central photon energy in the filtered white X-ray beam spectrum was around 40 keV; the voxel size was (3.07 μm)³. For each scan, 1800 projections were collected at sample orientation angles equally distributed over 180°; the acquisition time for each projection was 35 ms. The resulting reconstructed volume from each scan had a diameter and height of 2048 pixels, i.e., 6.3 mm. Three or four CT scans with approximately 750 μm overlap in the vertical direction were performed on each stent to cover the entire sample.

**Fig. 1:**
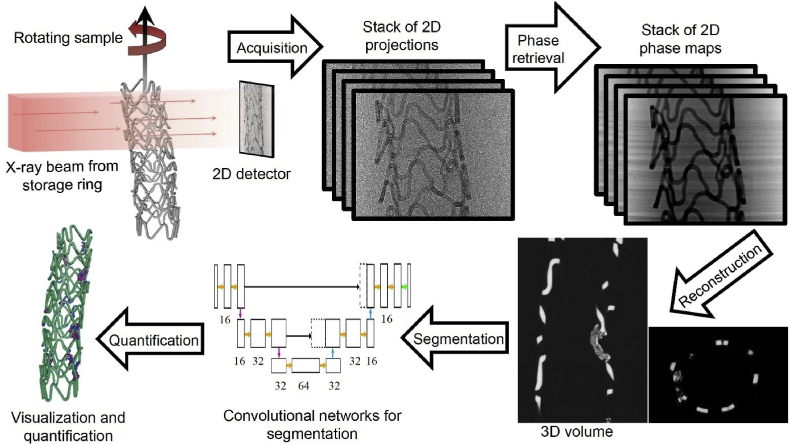
Illustrating the workflow from μCT data collection, phase retrieval and reconstruction, and image analysis.

Tomographic reconstruction was performed using the standard data processing pipeline of the ANATOMIX beamline, with a Python-based configuration tool written at SOLEIL and calling the reconstruction program PyHST2 developed at the European Synchrotron Radiation Facility (ESRF) [29]. A phase-retrieval filter of Paganin type [30] was used, with the parameter “Paganin length” in PyHST2 set to 107 μm. The reconstructed volume data from the individual scans of each sample were merged into one single file and converted into 16-bit integer values (see Fig. 1).

### 2.5 Synchrotron μCT data analysis

**Fig. 2:**
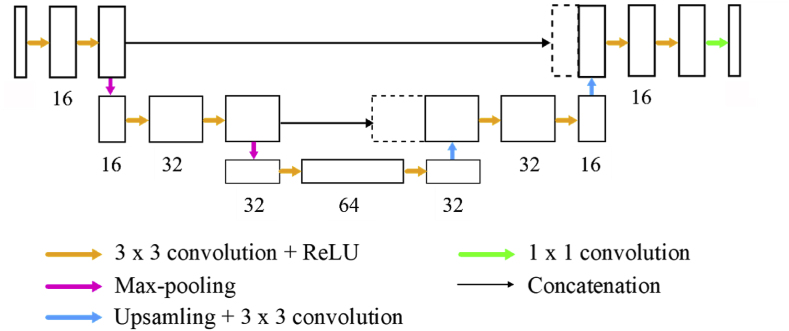
Illustration of the 2D U-Net architecture similar to Ronneberger et al. [18] The 2D U-Net used in this paper consists of three encoding stages. 3 × 3 convolutional filters were used, followed by non-linear activations (ReLU) and down-sampling by max-pooling. The number below each convolutional block denotes the number of feature maps. In the decoding stage, the volume was up-sampled by up-convolution. Finally, by a 1 × 1 convolution, the number of feature maps was reduced to 1 again. Skip connections between the encoding and the decoding path were realized by concatenations.

To evaluate the degradation volume of the Resoloy scaffolds, each volume was segmented into three different material compartments, namely into the metallic Mg phase, degraded Mg, and severely degraded Mg phase. The computer code for the segmentation was developed inhouse in the Python programming language, using the U-Net convolutional network (Fig. 2) [18].

A 2D U-Net with three encoding stages and two convolutions in each stage was used. The number of feature maps in the initial encoding stage was 16 and was doubled after each encoding stage. In total, this resulted in 186,409 trainable parameters for the network. A non-linear ReLU activation was applied after each convolution and max-pooling was used to down-sample the feature maps after each encoding stage. Up-sampling in the symmetric decoding path was achieved by up-convolution and the skip-connections between the corresponding encoding and decoding stages by concatenations. Finally, a 1 × 1 convolution reshaped the output to the same shape as the input. The network architecture is illustrated in Fig. 2.

The 3D information of the μCT volumes was partially preserved by extracting 2D patches from three orthogonal planes, namely the x-y, y-z and x-z planes. This resulted in 2400 training data pairs for one volume of size 800 × 800 x 800 when a patch size of 800 × 800 with zero overlap was used. In total, six of these volume pairs were used, resulting in 14,400 2D training data pairs. Training was carried out on an NVIDIA RTX A6000 graphics card with 48 GB GPU memory.

**Fig. 3:**
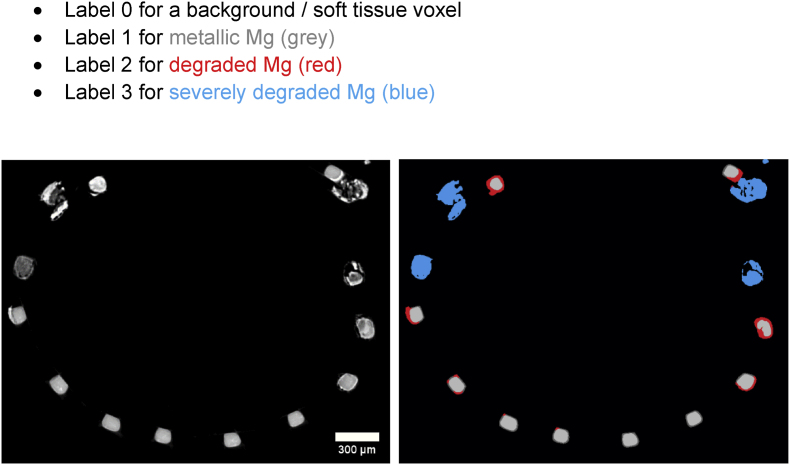
Illustration of the performance of the segmentation: Virtual cross section through the 3D image obtained through synchrotron μCT. Left: grey values as obtained by tomographic reconstruction. Right: segmented data, with metallic Mg in light grey, slightly degraded Mg in red, and severely degraded Mg in blue.

After segmentation, the 3D morphology of corroded and metallic Mg was quantified using Python. The surface area was computed in the software IPSDK (Reactiv IP, Grenoble France) which sums up the areas of the triangles forming the shape mesh of the segmented stents (Fig. 8, Appendix). This resulted in 4 labels for each sample (Fig. 3).

• Label 0 for a background/soft tissue voxel
• Label 1 for metallic Mg (grey)
• Label 2 for degraded Mg (red)
• Label 3 for severely degraded Mg (blue)

The visualization of the renderings (Fig. 6) were obtained with the AVIZO software (Thermo Fisher Scientific, Waltham, Massachusetts, USA).

### 2.6 Statistical analysis

All statistical analyses were performed using the statistics toolbox in MATLAB (MathWorks, Natick, Massachusetts, USA). Differences between four groups were assessed by analyses of variance (ANOVA), followed by post hoc multiple comparison Tukey-Kramer tests. The small number of samples per group analyzed by synchrotron μCT (N = 3) limits the power of the statistical evaluation. All statistical results were considered significant for *p* < 0.05.

## 3 Results

### 3.1 SEM and EDX

The elemental mapping qualitatively reveals the degradation of Resoloy in vivo.

Degradation occurs similarly for all groups: The struts are degrading from the surface to the inside. Degradation products consist mainly of calcium (Ca), phosphorus (P) and oxygen (O), while dysprosium (Dy) is present in the non-degraded area and also within the degradation products.

Primarily, in a slightly degraded state, the degradation products (Ca, O, P) do not enlarge the initial cross section area. These products seem to be brittle as some cracks appear during SEM analysis, which is not seen for the non-degraded material.

Degradation products of severely degraded struts occupy a larger area than the original strut material due to dismantling and larger cracks. In these large cracks the predominant element which can be found is carbon (Fig. 5).

**Fig. 4:**
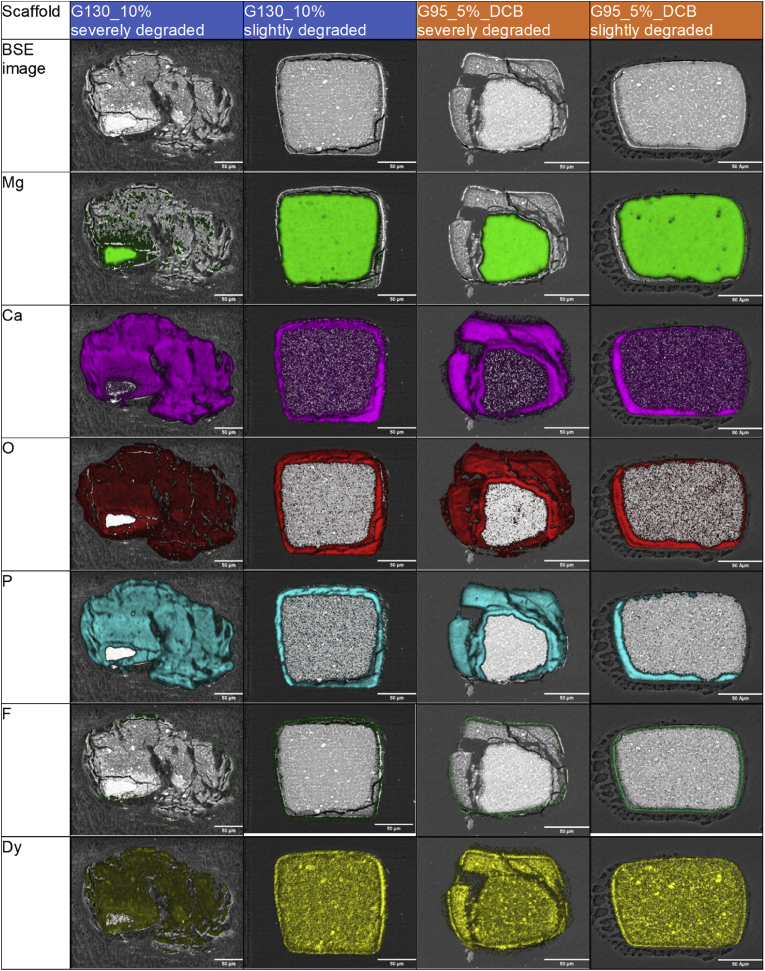
Illustration of the elemental distribution of two groups (G130_10% and G95_5%_DCB). For each group, one severely and one slightly degraded strut are shown. The detected elements (Mg, Ca, O, P, F and Dy) are depicted in different colors. Higher color intensity stands for more detected counts of this element. Scale bars: 50 μm.

**Fig. 5:**
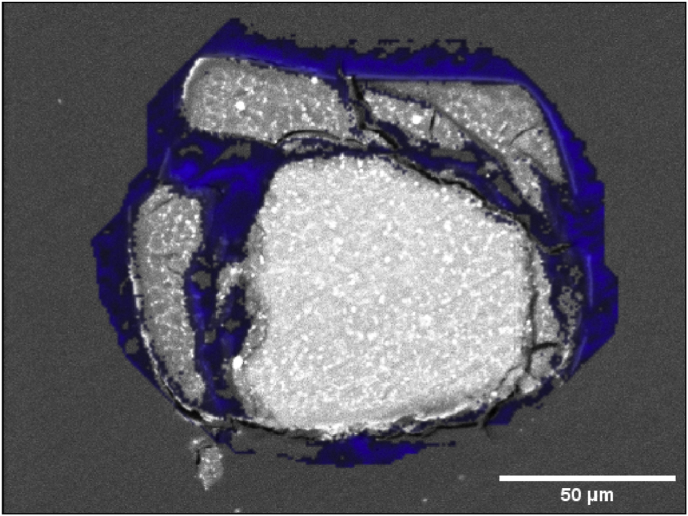
Carbon distribution (in blue) of a severely degraded strut according to G95_5%_DCB of Fig. 4.

**Fig. 6:**
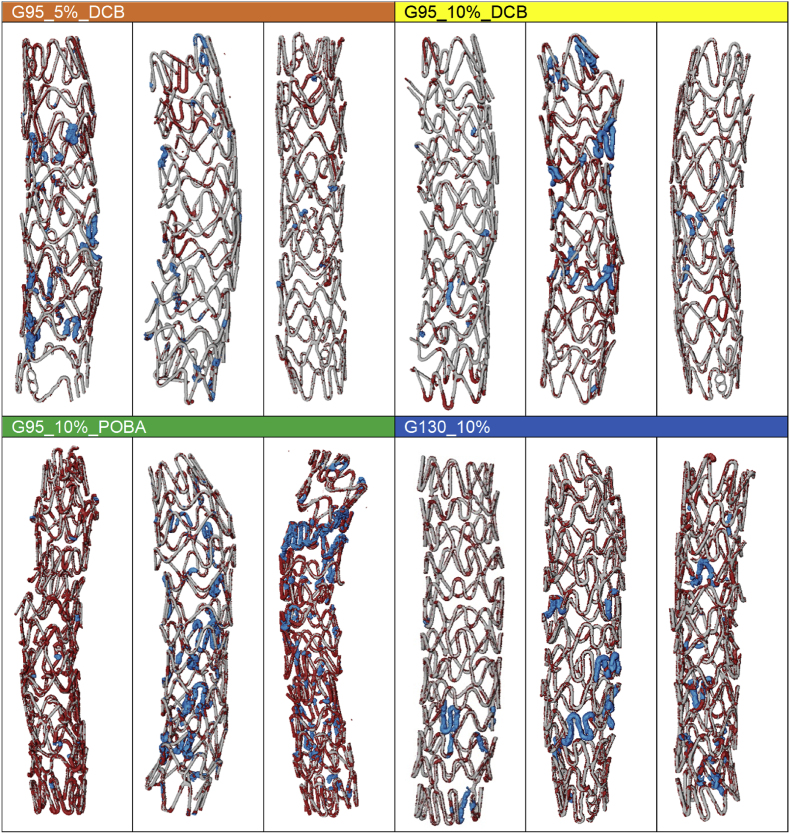
Segmented 3D-renderings of all scaffolds analyzed with SR-μCT. *The remaining Resoloy material, the degraded and the severely degraded regions, are marked, respectively, in grey, red, and blue*.

The same analysis was performed for the remaining groups and revealed qualitatively corresponding results (data not shown).

### 3.2 Synchrotron micro-CT (qualitative)

The 3D-rendered models reveal the shape and general appearance of all scaffolds (Fig. 6). The remaining Resoloy material, the degraded and the severely degraded regions, are marked, respectively, in grey, red, and blue. Animated versions of the rendered models can be found in the supplementary files.

Groups post-dilated with DCB appear to be less degraded than those post-dilated with POBA and the non-post-dilated ones. Mostly uniform and slight degradation is seen, while single scaffolds of each group appear severely degraded. Slight degradation occurs predominantly in highly bent scaffold regions but not in all of them, severely degraded regions are randomly distributed. Furthermore, the degradation products in severely degraded regions lose their initial strut shape and appear bulkier or broken up in parts. Group G95_10%_POBA shows the highest degree of degradation and apparently the highest loss of diameter, while group G95_5%_DCB appear to be mostly intact with mildly degraded parts.

### 3.3 Synchrotron micro-CT (quantitative)

**Fig. 7:**
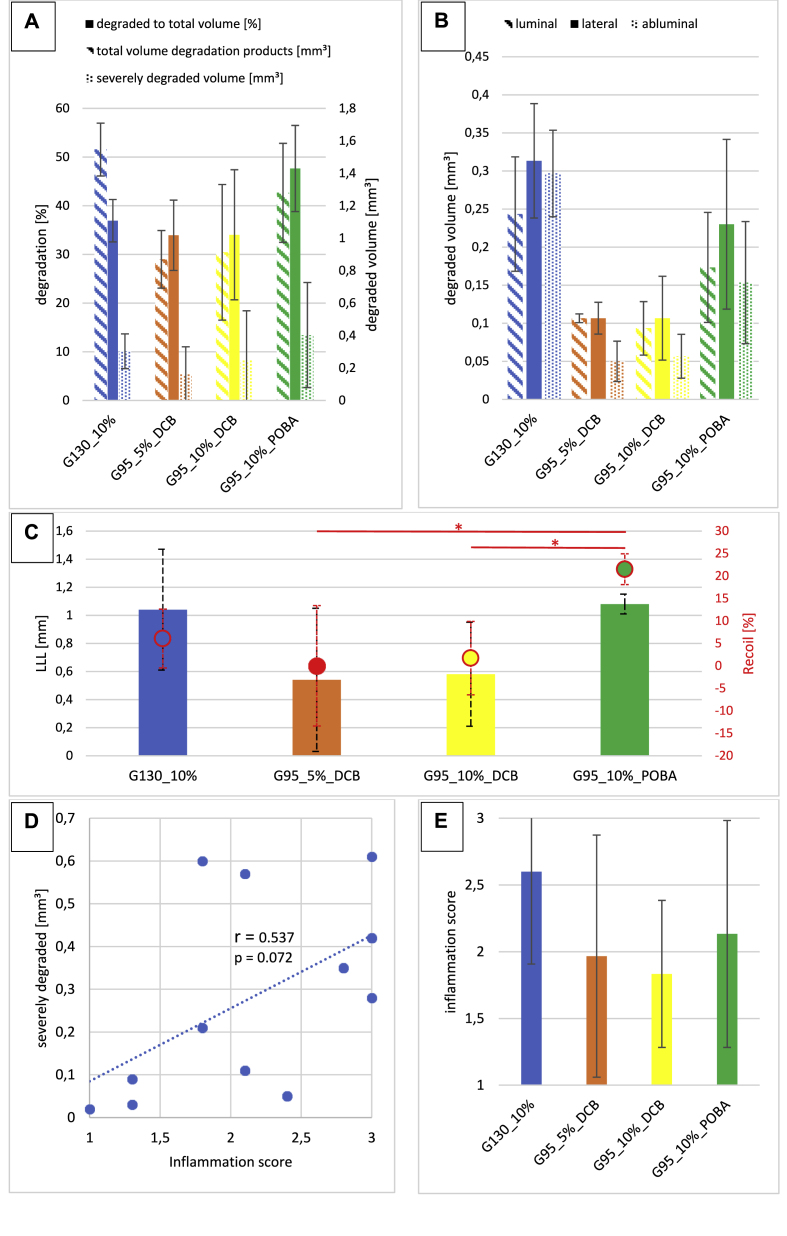
**A**: SR-μCT degradation analysis for all groups. Hatched bars: total volume of degraded material (right y-axis); filled bars: proportional degradation with respect to the total scaffold volume (left y-axis); dotted bars: volume severely degraded material (right y-axis). **B**: Spatially resolved degradation analysis of scaffold struts (see Fig. 8 of appendix). Hatched bars: luminal degradation volume; filled bars: lateral degradation volume; dotted bars: abluminal degradation volume. **C**: Results of Recoil and LLL derived from angiography for all groups. Bars: LLL values. Circles: Recoil values (error bars in red), statistically significant differences are marked with an asterisk (*). **D**: Correlation between the volumes of severely degraded scaffold-parts and the corresponding inflammation scores for each scaffold of all groups. **E**: Inflammation scores per scaffold-group.

**Fig. 8:**
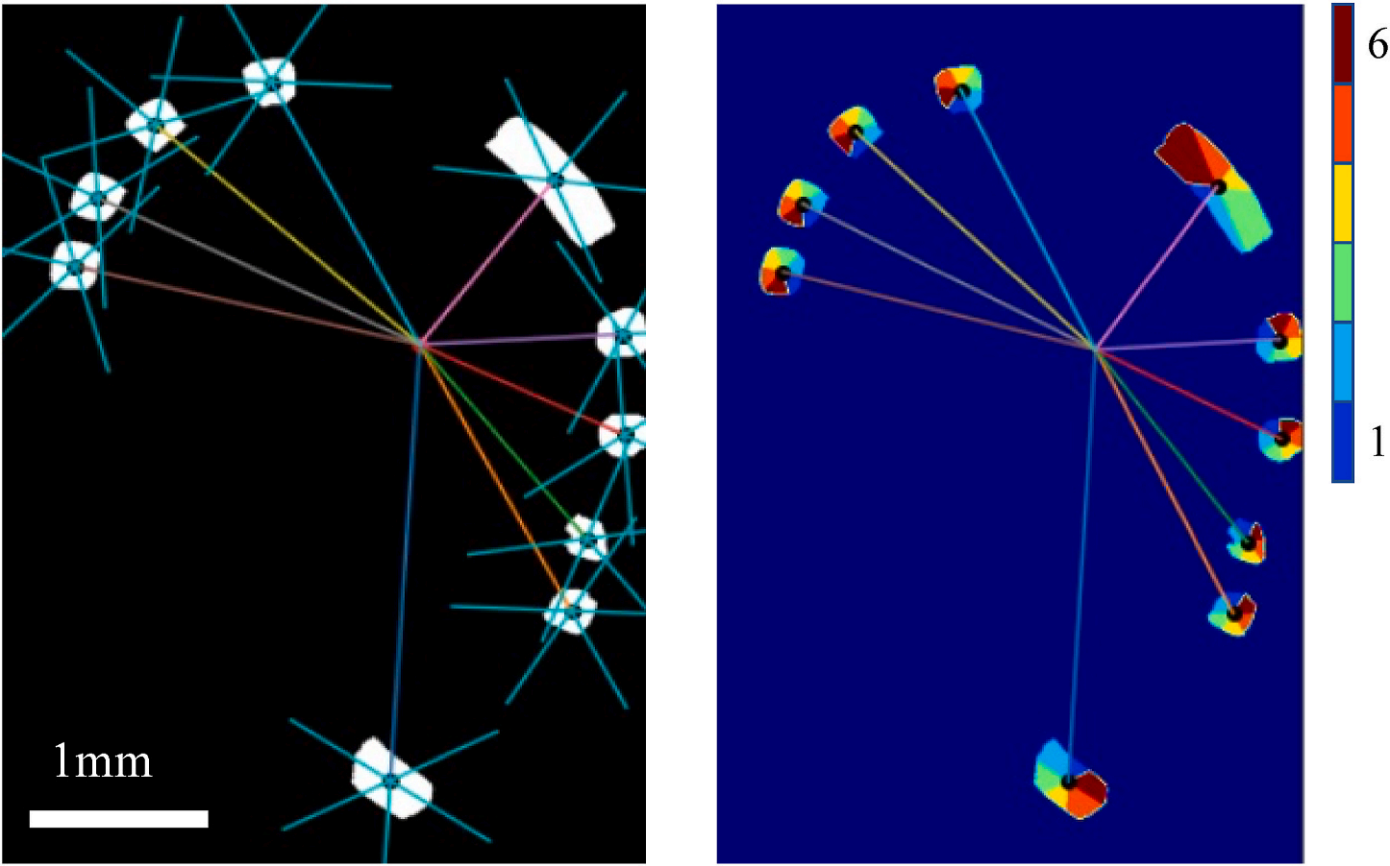
**Left**: Division of each strut into segments which have a certain orientation towards a reference point, calculated as the center of mass of all struts in the 2D plane. The lines connecting the reference point with the centers of mass of each individual struts crosses the region which is assumed as the luminal region. From this starting line, different segments are defined, which have the same angle range. **Right**: Example showing how each strut is divided into 6 different segments. Here, all segments having the numbers 1 and 2 are approximately in the luminal region whereas segments 5 and 6 are rather in the abluminal region. By using a smaller angle range, each strut can be divided into more segments, allowing a closer approximation to the luminal and abluminal regions. In this paper, each strut has been divided into 18 segments.

The analysis of the degraded material volume is summarized in Fig. 7 and suggests differences between the groups. Both groups post-dilated with DCB (G95_10%_DCB 0.91 mm³ and G95_5%_DCB 0.87 mm³) show a smaller amount of degraded material than the other groups without drug (G130_10% 1.54 mm³ and G95_10%_POBA 1.28 mm³). Also, the volumes of severely degraded material are smaller in the DCB groups than in the groups without drug. If the fractions of degraded material to total volume of the scaffolds are compared, the same tendencies are evident, with the notable exception of group G130_10%. This latter group has thicker struts and therefore a larger total volume.

By segmenting each strut into luminal, lateral and abluminal areas, the differences in degradation between these different locations are disclosed (Fig. 7 B). The totally degraded areas (marked in blue in Fig. 3, Fig. 6) are excluded for this comparison and the mildly degraded areas (Fig. 3, marked in red) are analyzed.

While the scaffolds without drug treatment seem to degrade preferentially from the lateral and abluminal areas, the DCB post-dilated ones preferentially degrade luminally and from the sides. There is less degradation for the abluminal areas. The non-drug groups exhibit 3–6 times higher volumetric degradation when the abluminal areas are compared. The ratios of luminal to abluminal degraded volume of all six DCB samples (min = 1.4, max = 3.3) are higher than all six POBA samples (min = 0.7, max = 1.3), see Table 3 in Appendix.

### 3.4 OCT and QCA

Both the recoil and the LLL are reduced for the DCB post-dilated scaffolds compared to POBA and without post-dilatation after 28 d (Fig. 7C). There are no notable differences between G95_5%_DCB and G95_10%_DCB. While the LLL values are equal between G130_10% and G95_10%_POBA, the recoil is larger for the latter.

### 3.5 Inflammation score and severe degradation

The inflammation scores from histology show less tissue inflammation for the DCB groups (Fig. 7 E). There is a positive correlation between the volume of severely corroded scaffold parts and the inflammation score of each scaffold (Fig. 7 D).

## 4 Discussion

Our previous study showed the safe application of Resoloy scaffolds in the coronary arteries with good clinical outcomes without any drugs and strut thicknesses of 130 μm [31]. In this study, we shed light on the degradation process of Resoloy scaffolds with thinner struts and the influence of paclitaxel, administered during post-dilatation with a DCB. As a reference base, we implanted the mentioned scaffolds with thicker struts (G130_10%).

The occurring elements during degradation were inspected by SEM and EDX. The degradation layers consist mainly of C, P and O. Dy and F are remaining in the degradation products due to their poor solubility in water. Comparing all groups, there are no obvious notable differences: the degradation products and product appearance are basically identical, even for the scaffolds with a smaller content of Dy (G95_5%_DCB). This degradation course can be described as the evolution of hydroxides and phosphates to calcium phosphates doped with less soluble secondary phases rich in rare earths. The detected C in the gaps of the cracked degradation products (Fig. 5) is in line with the observation of infiltrating cells in these areas [31]. Immune cells might be responsible for the further dissolution of degradation products, which is shown for comparable Mg-alloys [[31], [32], [33], [34], [35]].

SR-μCT and SEM analysis reveal two types of degradation of the scaffolds: slight and severe, localized degradation. The slight degradation occurs predominantly in the bends and plastically deformed parts of the scaffold, which may be stress-induced. This corresponds to the in vitro degradation of magnesium scaffolds but it cannot be referred to the severe, localized degradation [31,[36], [37], [38], [39], [40]]. There is only scarce literature dealing with the influence of anti-proliferative drugs on the degradation rates of magnesium alloys. It can be speculated that the biological response plays a decisive role for the degradation of magnesium alloys as inflammatory cells are known to elute acidic cocktails and are capable of phagocytosis of degradation products [34,41,42]. Thus, the presence of immune cells could provoke severe localized degradation. As paclitaxel acts as a cell inhibitor, also for immune cells, this could be a reason for less severe, localized degradation of DCB post-dilated scaffolds than with POBA or without post-dilatation [43,44]. The correlation of inflammation score and severe degradation (Fig. 7 D) might be evidence for this hypothesis but verification in further studies is necessary.

Apart from the biological influence on degradation, the processing of the magnesium scaffolds plays an important role. In this study, we used extruded micro-tubes with a fine microstructure and relatively high ultimate tensile strength and ductility as a scaffold base material, which enables thin struts with good radial resistance. The passivation layer and the fine microstructure could prevent the scaffold from severe degradation, as was demonstrated by many researchers for a broad range of magnesium alloys in the past [31,42,[45], [46], [47], [48], [49]]. Anyway, as for this study we used for all scaffolds the same processing route from melting to laser cutting, we would not assume a variation of degradation due to microstructure differences. Apart from the strut thicknesses, the scaffold designs were identically, which also promotes comparability.

Further, we can conclude that the wall thickness of the scaffolds (G95_10%_POBA vs. G130_10%) could also influence the degradation rate (Fig. 7). A reduced wall thickness might accelerate the embedding in the vessel wall and provoke less inflammatory response [21,50,51]. Both would be beneficial in terms of degradation speed: the former reduces the time of the scaffold material in the blood flow and the latter reduces the risk of the mentioned biological influence. The same hemodynamic mechanism could hold true to explain the differences of abluminal and luminal degradation speed of DCB post-dilated scaffolds (Fig. 7 B). The luminal areas of these scaffolds are in the blood flow for a longer time, due to the administration of paclitaxel, which is hindering cells to cover the struts. Without DCB post-dilatation, scaffolds are colonized faster by cells, which might explain the lower luminal degradation compared to lateral and abluminal for this group - although the degradation is higher compared to the DCB groups [34,41,42,[52], [53], [54]]. It should be mentioned that the lateral degradation volumes are slightly overestimated as the scaffold struts are rectangular with a larger width than thickness but there are two lateral sides included. Further studies with a larger number of samples would be necessary to corroborate these hypotheses.

The OCT and QCA results of recoil and LLL are in line with the results of the degradation rates: DCB post-dilated scaffolds show a smaller LLL and recoil due to smaller degradation rates than POBA post-dilated. While the LLL could also be induced by a thicker neointima formation, the recoil is induced by degradation [[55], [57], [56]]. In particular, the recoil corresponds well with the severe, localized degradation, which acts detrimentally on the radial force of a scaffold. The smaller recoil value of G130_105_POBA could be explained by the thicker strut sizes of the scaffolds, which compensates the degradation mechanically.

**Fig. 9:**
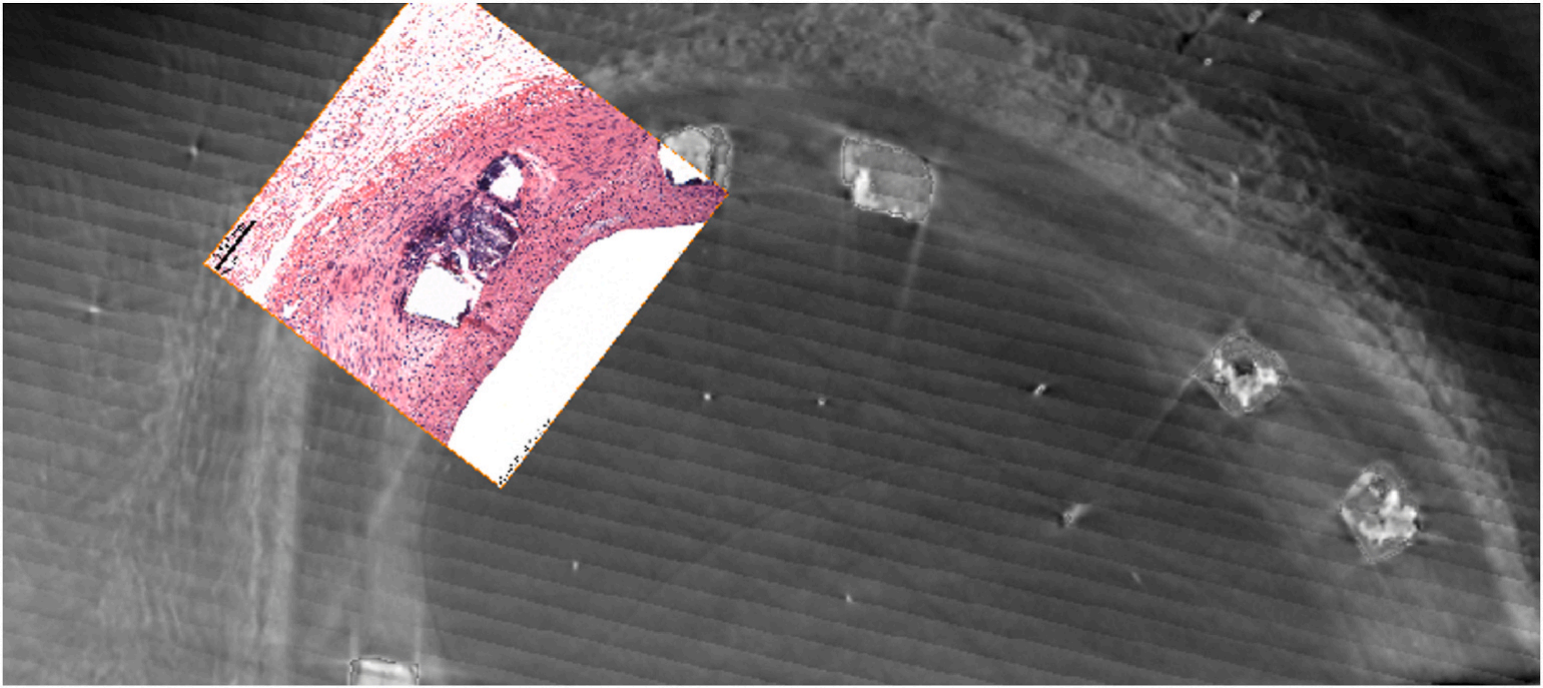
Overlay of a laboratory processed histology image (colored) and the 2D-image of the SRμ-CT (grey).Please note, that the contrast has been adjusted to show the grey-values corresponding to the soft tissue.

SR-μCT in combination with advanced image processing methods is a game changer in the degradation analysis of Mg-based implants due to the fast data collection, high sensitivity, and high resolution. This study shows the feasibility of using this technique also for smallest cardiovascular scaffolds. While laboratory μCT can reveal macroscopic defects like strut discontinuities and qualitatively degradation areas, with SR-μCT even different degradation products can be distinguished. The high image contrast and spatial resolution enable quantitative examination of the degradation. Moreover, it is possible to evaluate the soft tissue surrounding the scaffold struts (Fig. 9, Appendix) and calculate morphometric values like stenosis [%], neointima area [mm^2^], etc. on thousands of slices as opposed to the standard histology reference of three slices per stent/scaffold. DL-powered image processing together with dedicated analysis pipelines enables fast and highly automated data analysis beyond currently accessible material properties – for example, to study the degradation in different regions of each strut, such as in the luminal versus abluminal region.

## 5 Conclusion

Post dilatation of Resoloy scaffolds with DCBs as well as thinner struts appear to enhance clinical outcomes like LLL and recoil. We hypothesize that this is due to a prolonged and more homogenous degradation compared to thicker scaffolds without a drug treatment in vivo. After 28 days in vivo, the scaffolds were degraded by 30–50 vol.-%, depending on the treatment method and strut thickness. Inflammation score and localized, severe degradation are positively correlated to each other. The administration of anti-inflammatory drugs could enhance the time of radial stability of magnesium scaffolds.

Furthermore, this study confirms the outstanding performance of SR-μCT in combination with data analysis powered by deep learning (DL) to evaluate the degradation morphology quantitatively as well as qualitatively with a high spatial resolution. Additionally, this method holds promise for soft tissue morphology evaluation in future investigations.

## Ethics_approval

The tests were performed at the Institute of Medical Technology and Research (IMTR GmbH, Rottmersleben, Germany). All animals received standard care outlined in accordance with the EU Commission Directive 86/609/EEC and the German Animal Protection Act based upon the Animal Ethics Committee approvals (Saxony-Anhalt, Germany).

## Declaration of competing interest

R. Menze is an employee of MeKo Manufacturing e.K., B. Hesse is CEO and shareholder of Xploraytion GmbH, D. Chen was an employee of Xploraytion GmbH, M. Kusmierczuk is an employee and B. Scheller is a shareholder of InnoRa GmbH, T. Weitkamp and S. Bettink have no conflicts of interest to declare.

## Figures and Tables

**Table 3:** Summary of the computed morphological properties.

group	sample	total volume [mm³]	metallic phase [mm³]	all degraded phases [mm³]	severely degraded Phase [mm³]	% degraded to total	lum. degra. vol. [mm³]	ablum. degra. vol. [mm³]	lateral degra. vol. [mm³]	ratio luminal/abluminal corrosion volume
G95_5%_DCB	G136-30-LCX	2.45	1.72	0.73	0.02	29.80	0.11	0.04	0.1	2.75
	G136-34-RCA	2.72	1.91	0.81	0.11	29.78	0.1	0.03	0.09	3.33
	G136-35-LAD	2.53	1.45	1.07	0.35	42.29	0.11	0.08	0.13	1.38
G95_10%_DCB	G136-32-RCA	2.63	1.81	0.82	0.09	31.18	0.09	0.04	0.08	2.25
	G136-34-LCX	2.82	1.45	1.37	0.6	48.58	0.13	0.09	0.17	1.44
	G136-35-RCA	2.46	1.91	0.55	0.05	22.36	0.06	0.04	0.07	1.50
G95_10%_POBA	G136-31-LAD	2.41	1.44	0.97	0.03	40.25	0.21	0.16	0.25	1.31
	G136-32-LCX	2.85	1.56	1.29	0.57	45.26	0.09	0.07	0.11	1.29
	G136-35-LCX	2.75	1.17	1.58	0.61	57.45	0.22	0.23	0.33	0.96
G130_10%	G136-26-LAD	4.25	3.93	1.36	0.21	32.00	0.32	0.36	0.39	0.89
	G136-27-LAD	4.21	3.97	1.62	0.42	38.48	0.24	0.28	0.31	0.86
	G136-29-LAD	4.12	3.95	1.66	0.28	40.29	0.17	0.25	0.24	0.68

**Table 4:** In vivo test data.

group	sample	recoil 28 d [%]	LLL [mm]	inflammation score
G95_5%_DCB	G136-30-LCX	5.95	–	1.0
	G136-31-RCA	2.06	0.28	2.0
	G136-32-LAD	14.07	1.31	2.3
	G136-33-LCX	–	–	2.0
	G136-34-RCA	−21.79	0.25	2.1
	G136-35-LAD	−3.15	0.32	2.8
G95_10%_DCB	G136-30-LAD	12.15	–	1.4
	G136-31-LCX	−3.90	0.62	1.9
	G136-32-RCA	8.75	1.04	1.3
	G136-33-LAD	–	–	2.5
	G136-34-LCX	−4.10	0.49	1.8
	G136-35-RCA	−4.87	0.15	2.4
G95_10%_POBA	G136-30-RCA	20.80	–	1.7
	G136-31-LAD	17.16	0.98	1.3
	G136-32-LCX	–	1.07	2.1
	G136-33-RCA	–	–	2.6
	G136-34-LAD	25.20	1.14	2.5
	G136-35-LCX	22.87	1.11	3.0
G130_10%	G136-24-LAD	9.24	1.47	1.6
	G136-24-LCX	14.74	0.71	3.0
	G136-25-RCA	−0.78	0.98	2.6
	G136-25-LCX	8.62	1.58	3.0
	G136-26-RCA	0.61	0.45	1.5
	G136-26-LAD	1.33	0.89	1.8
	G136-27-LAD	3.72	0.79	3.0
	G136-27-LCX	4.07	0.31	2.3
	G136-28-RCA	7.28	1.45	2.3
	G136-28-LCX	−5.22	1.05	1.6
	G136-29-RCA	11.37	1.44	3.0
	G136-29-LAD	16.77	1.37	3.0

## References

[1] Lipinski M.J., Acampado E., Cheng Q., Adams L., Torii S., Gai J., Torguson R., Hellinga D.G., Joner M., Harder C., Zumstein P., Finn A.V., Kolodgie F.D., Virmani R., Waksman R. (2019). Comparison of acute thrombogenicity for magnesium versus stainless steel stents in a porcine arteriovenous shunt model. EuroIntervention.

[2] Joner M., Ruppelt P., Zumstein P., Lapointe-Corriveau C., Leclerc G., Bulin A., Castellanos M.I., Wittchow E., Haude M., Waksman R. (2018). Preclinical evaluation of degradation kinetics and elemental mapping of first- and second-generation bioresorbable magnesium scaffolds. EuroIntervention.

[3] Serruys P.W., Chevalier B., Sotomi Y., Cequier A., Carrié D., Piek J.J., Van Boven A.J., Dominici M., Dudek D., McClean D., Helqvist S., Haude M., Reith S., de Sousa Almeida M., Campo G., Iñiguez A., Sabaté M., Windecker S., Onuma Y. (2016). Comparison of an everolimus-eluting bioresorbable scaffold with an everolimus-eluting metallic stent for the treatment of coronary artery stenosis (ABSORB II): a 3 year, randomised, controlled, single-blind, multicentre clinical trial. Lancet.

[4] Verheye S., Wlodarczak A., Montorsi P., Torzewski J., Bennett J., Haude M., Starmer G., Buck T., Wiemer M., Nuruddin A.A.B., Yan B.P.Y., Lee M.K.Y. (2020).

[5] Haude M., Toelg R., Lemos P.A., Christiansen E.H., Abizaid A., von Birgelen C., Neumann F.-J., Wijns W., Ince H., Kaiser C., Lim S.T., Escaned J., Eeckhout E., Garcia-Garcia H.M., Waksman R. (2022). Sustained safety and performance of a second-generation sirolimus-eluting absorbable metal scaffold: long-term data of the BIOSOLVE-II first-in-man trial at 5 years. Cardiovasc. Revascularization Med..

[6] Seguchi M., Baumann-zumstein P., Fubel A., Waksman R. (2023).

[7] Hijazi Z.M. (2015).

[8] Alexy R.D., Levi D.S. (2013).

[9] Zartner P.A., Schranz D., Mini N., Schneider M.B. (2020).

[10] Zeller-Plumhoff B., Helmholz H., Feyerabend F., Dose T., Wilde F., Hipp A., Beckmann F., Willumeit-Römer R., Hammel J.U. (2018). Quantitative characterization of degradation processes in situ by means of a bioreactor coupled flow chamber under physiological conditions using time-lapse SRμCT. Mater. Corros..

[11] Feyerabend F., Dose T., Xu Y., Beckmann F., Stekker M., Willumeit-Römer R., Schreyer A., Wilde F., Hammel J.U. (2016). Magnesium degradation observed in situ under flow by synchrotron radiation based microtomography. Dev X-Ray Tomogr X.

[12] Gonzalez J., Hou R.Q., Nidadavolu E.P.S., Willumeit-Römer R., Feyerabend F. (2018). Magnesium degradation under physiological conditions – best practice. Bioact. Mater..

[13] Pfeiffer F., Weitkamp T., Bunk O., David C. (2006). X-ray sources.

[14] Kačarević Ž.P., Rider P., Elad A., Tadic D., Rothamel D., Sauer G., Bornert F., Windisch P., Hangyási D.B., Molnar B., Kämmerer T., Hesse B., Bortel E., Bartosch M., Witte F. (2022). Biodegradable magnesium fixation screw for barrier membranes used in guided bone regeneration. Bioact. Mater..

[15] Rider P., Kačarević Ž.P., Elad A., Rothamel D., Sauer G., Bornert F., Windisch P., Hangyási D., Molnar B., Hesse B., Assad M., Witte F. (2022). Biodegradation of a magnesium alloy fixation screw used in a guided bone regeneration model in beagle dogs. Materials.

[16] Langer M., Cloetens P., Hesse B., Suhonen H., Pacureanu A., Raum K., Peyrin F. (2014). Priors for X-ray in-line phase tomography of heterogeneous objects. Philos Trans R Soc A Math Phys Eng Sci.

[17] Cicek Ozgur, Ahmed Abdulkabdir, Soeren S. (2016). Lienkamp, thomas brox OR. 3D U_net. Med Image Comput Comput Interv.

[18] Navab N., Hornegger J., Wells W.M., Frangi A.F. (2015). Medical image computing and computer-assisted intervention - MICCAI 2015: 18th international conference Munich, Germany, october 5-9, 2015 proceedings, part III. Lect. Notes comput. Science.

[19] Jung O., Hesse B., Stojanovic S., Seim C., Weitkamp T., Batinic M., Goerke O., Ž Peri (2021).

[20] Baltruschat I.M., Ćwieka H., Krüger D., Plumhoff B.Z. (2021). Scaling the U - net : segmentation of biodegradable bone implants in high - resolution synchrotron radiation microtomograms. Sci. Rep..

[21] Hara H., Nakamura M., Palmaz J.C., Schwartz R.S. (2006).

[22] Bønaa K.H., Mannsverk J., Wiseth R., Aaberge L., Myreng Y., Nygård O., Nilsen D.W., Kløw N.-E., Uchto M., Trovik T., Bendz B., Stavnes S., Bjørnerheim R., Larsen A.-I., Slette M., Steigen T., Jakobsen O.J., Bleie Ø., Fossum E., Hanssen T.A., Dahl-Eriksen Ø, Njølstad I., Rasmussen K., Wilsgaard T., Nordrehaug J.E. (2016). Drug-eluting or bare-metal stents for coronary artery disease. N. Engl. J. Med..

[23] Waksman R., Lipinski M.J., Acampado E., Cheng Q., Adams L., Torii S., Gai J., Torguson R., Hellinga D.M., Westman P.C., Joner M., Zumstein P., Kolodgie F.D., Virmani R. (2017). Comparison of acute thrombogenicity for metallic and polymeric bioabsorbable scaffolds: Magmaris versus absorb in a porcine arteriovenous shunt model. Circ Cardiovasc Interv.

[24] Ali Z.A., Gao R., Kimura T., Onuma Y., Kereiakes D.J., Ellis S.G., Chevalier B., Vu M.T., Zhang Z., Simonton C.A., Serruys P.W., Stone G.W. (2018). Three-year outcomes with the absorb bioresorbable scaffold. Circulation.

[25] Jinnouchi H., Torii S., Sakamoto A., Kolodgie F.D., Virmani R., Finn A.V. (2019). Fully bioresorbable vascular scaffolds: lessons learned and future directions. Nat. Rev. Cardiol..

[26] Kornowski R.A.N., Hong M.U.N.K., Tio F.O., Bramwell O., Wu H., Leon M.B. (1998). In-stent restenosis : contributions of inflammatory responses and arterial injury to neointimal hyperplasia. J. Am. Coll. Cardiol..

[27] Weitkamp T., Scheel M., Giorgetta J., Joyet V., Le Roux V., Cauchon G., Moreno T., Polack F., Thompson A., Samama J. (2017). The tomography beamline ANATOMIX at Synchrotron SOLEIL. J Phys Conf Ser.

[28] Weitkamp T., Scheel M., Perrin J., Daniel G., King A., Le Roux V., Giorgetta J.L., Carcy A., Langlois F., Desjardins K., Menneglier C., Cerato M., Engblom C., Cauchon G., Moreno T., Rivard C., Gohon Y., Polack F. (2022). Microtomography on the ANATOMIX beamline at synchrotron SOLEIL. J Phys Conf Ser.

[29] Mirone A., Brun E., Gouillart E., Tafforeau P., Kieffer J. (2014). The PyHST2 hybrid distributed code for high speed tomographic reconstruction with iterative reconstruction and a priori knowledge capabilities. Nucl. Instrum. Methods Phys. Res. Sect. B Beam Interact. Mater. Atoms.

[30] Scientific C., Science M. (2002).

[31] Menze R., Wittchow E. (2021). In vitro and in vivo evaluation of a novel bioresorbable magnesium scaffold with different surface modifications. J. Biomed. Mater. Res. Part B Appl Biomater.

[32] Zhang J., Li H., Wang W., Huang H., Pei J., Qu H., Yuan G., Li Y. (2018). The degradation and transport mechanism of a Mg-Nd-Zn-Zr stent in rabbit common carotid artery: a 20-month study. Acta Biomater..

[33] Li H., Zhong H., Xu K., Yang K., Liu J., Zhang B., Zheng F., Xia Y., Tan L., Hong D. (2011). Enhanced efficacy of sirolimus-eluting bioabsorbable magnesium alloy stents in the prevention of restenosis. J. Endovasc. Ther..

[34] Zhang J., Hiromoto S., Yamazaki T., Huang H., Jia G., Li H., Yuan G. (2017). Macrophage phagocytosis of biomedical Mg alloy degradation products prepared by electrochemical method. Mater. Sci. Eng. C.

[35] Sefa S., Wieland D.C.F., Helmholz H., Zeller-Plumhoff B., Wennerberg A., Moosmann J., Willumeit-Römer R., Galli S. (2022). Assessing the long-term in vivo degradation behavior of magnesium alloys - a high resolution synchrotron radiation micro computed tomography study. Front Biomater Sci.

[36] Maier P., Griebel A., Jahn M., Bechly M., Menze R., Bittner B., Schaffer J. (2019). Corrosion bending fatigue of RESOLOY® and WE43 magnesium alloy wires. Miner Met Mater Ser.

[37] Maier P., Steinacker A., Clausius B., Hort N. (2020). Influence of solution heat treatment on the microstructure, hardness and stress corrosion behavior of extruded Resoloy. Jom.

[38] Maier P., Clausius B., Joy C., Menze R., Bittner B., Hort N., Miller V.M., Maier P., Jordon J.B., Neelameggham N.R. (2021). Magnes. Technol. 2021.

[39] Chen C., Chen J., Wu W., Shi Y., Jin L., Petrini L., Shen L., Yuan G., Ding W., Ge J., Edelman E.R., Migliavacca F. (2019). In vivo and in vitro evaluation of a biodegradable magnesium vascular stent designed by shape optimization strategy. Biomaterials.

[40] Wu W., Chen S., Gastaldi D., Petrini L., Mantovani D., Yang K., Tan L., Migliavacca F. (2013). Experimental data confirm numerical modeling of the degradation process of magnesium alloys stents. Acta Biomater..

[41] Wittchow E., Adden N., Riedmüller J., Savard C., Waksman R., Braune M. (2013). Bioresorbable drug-eluting magnesium-alloy scaffold: design and feasibility in a porcine coronary model. EuroIntervention.

[42] Schilling T., Brandes G., Tudorache I., Cebotari S., Hilfiker A., Meyer T., Biskup C., Bauer M., Waldmann K.H., Bach F.W., Haverich A., Hassel T. (2013). In vivo degradation of magnesium alloy LA63 scaffolds for temporary stabilization of biological myocardial grafts in a swine model. Biomed. Tech..

[43] Celletti F.L., Waugh J.M., Amabile P.G., Kao E.Y., Boroumand S., Dake M.D. (2002). Inhibition of vascular endothelial growth factor-mediated neointima progression with angiostatin or paclitaxel. J. Vasc. Intervent. Radiol..

[44] Wessely R., Schömig A., Kastrati A. (2006). Sirolimus and paclitaxel on polymer-based drug-eluting stents: similar but different. J. Am. Coll. Cardiol..

[45] Trivedi P., Misra R.D.K. (2018). Surface biodegradation behavior of rare earth- containing magnesium alloys with different microstructure: the impact on apatite coating formation on the surface. Mater. Technol..

[46] Trivedi P., Nune K.C., Misra R.D.K. (2016). Degradation behaviour of magnesium-rare earth biomedical alloys. Mater. Technol..

[47] Chen J., Yang Y., Etim I.P., Tan L., Yang K., Misra R.D.K., Wang J., Su X. (2021). Recent advances on development of hydroxyapatite coating on biodegradable magnesium alloys: a review. Materials.

[48] Trivedi P., Nune K.C., Misra R.D.K. (2017). Grain structure dependent self-assembled bioactive coating on Mg-2Zn-2Gd alloy: mechanism of degradation at biointerfaces. Surf. Coating. Technol..

[49] Witte F., Fischer J., Nellesen J., Vogt C., Vogt J., Donath T., Beckmann F. (2010). In vivo corrosion and corrosion protection of magnesium alloy LAE442. Acta Biomater..

[50] Foin N., Lee R.D., Torii R., Guitierrez-Chico J.L., Mattesini A., Nijjer S., Sen S., Petraco R., Davies J.E., Di Mario C., Joner M., Virmani R., Wong P. (2014). Impact of stent strut design in metallic stents and biodegradable scaffolds. Int. J. Cardiol..

[51] Jiménez J.M., Davies P.F. (2009). Hemodynamically driven stent strut design. Ann. Biomed. Eng..

[52] Liu D., Hu S., Yin X., Liu J., Jia Z., Li Q. (2018). Degradation mechanism of magnesium alloy stent under simulated human micro-stress environment. Mater. Sci. Eng. C.

[53] Wang J., Smith C.E., Sankar J., Yun Y., Huang N. (2015). Absorbable magnesium-based stent: physiological factors to consider for in vitro degradation assessments. Regen Biomater.

[54] Wang J., Giridharan V., Shanov V., Xu Z., Collins B., White L., Jang Y., Sankar J., Huang N., Yun Y. (2014). Flow-induced corrosion behavior of absorbable magnesium-based stents. Acta Biomater..

[55] Cubero-Gallego H., Vandeloo B., Gomez-Lara J., Romaguera R., Roura G., Gomez-Hospital J.A., Cequier A. (2017). Early collapse of a magnesium bioresorbable scaffold. JACC Cardiovasc. Interv..

[56] Marynissen T., McCutcheon K., Bennett J. (2018). Early collapse causing stenosis in a resorbable magnesium scaffold. Cathet. Cardiovasc. Interv..

[57] Yang H., Zhang F., Qian J., Chen J., Ge J. (2018). Restenosis in Magmaris stents due to significant collapse. JACC Cardiovasc. Interv..

